# The Effects of Leadership and Reward Policy on Employees’ Electricity Saving Behaviors: An Empirical Study in China

**DOI:** 10.3390/ijerph17062019

**Published:** 2020-03-18

**Authors:** Zhenjiao Chen, Yaqing Liu

**Affiliations:** School of Management and Economics, Beijing Institute of Technology, Beijing 100081, China; lyq_1027@126.com

**Keywords:** charismatic leadership, electricity saving behavior, responsibility, organizational reward policy

## Abstract

Excessive energy consumption and carbon emission damages the ecological environment and socially sustainable development in China. Organizations are major energy consumers, and should be principally responsible for energy saving. Despite that many organizational leaders know it is urgent to manage the energy saving behavior of employees, “how to effectively manage” receives less attention in both academic and practical fields. To fill this gap, this study adapts charismatic leadership theory to develop a theoretical model and explores how organizational leaders manage the electricity saving behavior of employees. This model was tested with a survey of 627 full-time employees from 14 provinces and municipalities of China. Results show that sustainable development vision, electricity saving cues, inspiration and role modeling significantly increase the electricity saving responsibility of employees, which in turn positively influences their electricity saving behavior. Moreover, organizational reward policy buffers the positive relationship between responsibility and behavior. This study contributes to energy conservation literature by explaining what characteristics of leadership improve the electricity saving behavior of employees and how leader characteristics match with organizational policy to effectively manage this. The practical implications for electricity saving management are also discussed.

## 1. Introduction

As global economics grows rapidly, one of the negative aspects is the over consumption of energy that leads to global warming and damages the ecological environment [1,2]. China is also facing an energy over-consumption problem [3,4]. In 2018, the energy production of China was 3.77 billion tons of standard coal equivalent, less than the recorded value in 2017. However, the energy consumption of China continued to increase, reaching as high as 4.64 billion tons of standard coal equivalent [5]. In addition to the environmental impact, energy consumption has long-term implications for health expenditure [6]. To inhibit damage to the environment, the United Nations (UN) required its 189 member states to frame their agendas from January 2016 until the end of 2030 around Sustainable Development Goals (SDGs). Sustainable development emphasizes economic growth in a manner that complies with environmental protection [7]. China, as a member of the UN, should commit to exploring effective energy saving methods and improving energy efficiency to realize SDG.

A literature review shows that energy saving was studied in both household and workplace contexts [4,8,9,10]. Research results relating to household energy saving cannot be generalized to the organizational context [4,8,11]. The twofold reason is that individual energy saving in an organization is not only affected by individual factors, but also promoted by organizational factors (such as organizational structure, norm, and climate) [8]. Moreover, the individual consumption costs of energy usage in a workplace differ from those in a household [4]. Extant research paid more attention to household energy saving than organizational energy saving. However, in reality, organizations (such as governments and enterprises) consume more energy than families. Organizations are the most accountable for environmental protection and socially sustainable development [12]. Electricity is a major form of energy consumed by various organizations Statistical reports show that organizational electricity usage accounts for over 85% of the total electricity usage in 2019 [13]. Researchers indicate that organizational electricity conservation is limited by the wasteful behavior of employees because they do not have to pay for their electricity usage in organizations [8,14]. A branch of research [4,15,16] only focuses on electricity saving issues of office workers in an office setting. For example, Zhang and colleagues (2013, 2014) used survey data collected from office workers in Beijing to test a theoretical model and found that social benefits, personal benefits, personal norms and organizational electricity saving climate significantly improve the electricity saving attitude and behavior of office workers in office settings [4,16].

They explained that they used office workers as a sample for two reasons. First, organizations in service industry, such as government institutions, schools, hospitals, financial companies, software companies, and other service enterprises do not engage in manufacturing. Employees in these organizations are office workers and electricity is the main form of energy they consumed in the workplace. Nowadays, there are more and more service organizations, which consume a lot of electricity every year. It is necessary to explore how to improve office workers can save electricity for their organizations. In order to include employees in these organizations as research objects, they took office workers as the sample. Second, the form of electricity saving behavior of office workers differs from that of other types of workers (e.g., production workers). For the same reasons, this study follows this branch of research to examine electricity saving behavior of office workers (hereafter referred to as “employees”) in office settings.

Studies have found that the energy saving attitude or behavior of employees can be encouraged by various organizational determinants, such as subjective norms, goal setting, individual feedback, group discussions, and rewards [8,16,17,18,19,20,21,22]. Leadership is inevitable in an organization and plays a pivotal role in influencing the attitudes and behaviors of employees [23]; however, leadership has received minimal attention in energy management research [8]. To fill this gap, we examine how leadership affects employees’ energy saving behaviors. Employees’ energy saving behaviors are typical extra-role behaviors, which refers to employees’ discretionary actions that go beyond their existing role expectations and are not mandated by their organizations [24]. In general, extra-role behaviors can be promoted by employees’ intrinsic motivation, which is more likely to be elicited by leader charisma. In leadership literature, researchers find that, compared with other leadership styles, charismatic leadership is more likely to improve employees’ intrinsic motivation and extra-role behaviors, such as organizational citizenship behaviors (OCBs) (i.e., discretionary behaviors [25], helping behaviors [26]). Although employees’ energy saving behavior is a typical extra-role behavior, the extant research into charismatic leadership pay little attention to it. To bridge the gap between charismatic leadership literature and energy management literature, we establish a theoretical model based on charismatic leadership theory (CLT) to demonstrate how the behavior of a charismatic leader improves employee electricity saving behavior, a typical extra-role behavior.

CLT highlights that charismatic leadership, as an organizational intervention, improves employee electricity saving behaviors by driving employee intrinsic motivation (e.g., a sense of responsibility). However, this theory ignores the effect of extrinsic motivation. In reality, employee extrinsic motivations and intrinsic motivations could be affected by organizational interventions simultaneously, and employee energy saving behaviors could be influenced by both of the two types of motivation [11,16]. We integrate extrinsic motivation literature [27] with CLT, and explore the moderating effect of organizational reward policy (i.e., a stimulus of extrinsic motivation) in our research model. A survey was conducted and data was collected from full-time office workers in China to test the theoretical model.

Section 2 provides a comprehensive literature review and the theoretical background. Section 3 presents the research model and develops the hypotheses. Section 4 describes the research method. Section 5 reports the data analysis and findings. Section 6 discusses the theoretical and practical implications of the study. Section 7 concludes the study. 

## 2. Literature Review and Theoretical Background

### 2.1. Electricity Saving in the Workplace 

A thorough review of the literature on organizational energy conservation shows that the determinants of energy saving attitude and behavior are divided into two categories: individual and organizational determinants [8,11,16]. One line of research proposes that individual level predictors, such as individual perceptions, abilities, attitudes, moral obligations, personalities, and past behavior are the main factors that drive employees to engage voluntarily in energy saving behavior [11,28,29]. Chen and Knight (2014) adopted the theory of planned behavior to show that perceived behavioral control and energy concern positively affect the energy conservation intention of employees in Chinese electric power companies [28]. The empirical study of Zhang et al. (2013) found that moral obligation, awareness of consequence, and ascription of responsibility positively affect the electricity saving behavior of employees of service organizations in Beijing [4]. Leygue et al. (2017) demonstrated that organizational identification, commitment, and motivation to help improve the image of one’s organization significantly increase the energy saving behavior of employees in their workplace [11]. Cordano and Frieze (2000) found that past behavior has a positive relationship with environmental managers’ preference for implementing future source reduction activities [29]. 

Another line of research asserts that the electricity saving attitude and behavior of employees can be influenced by organizational factors, including social norms, organizational climate, pro-environmental policy, goal setting, rewards, and group discussions [11]. Zhang et al. (2014) found that environmental benefits, organizational benefits, and social norms (i.e., the viewpoints of colleagues regarding electricity conservation) considerably affect the electricity saving attitude and behavior of employees [16]. Dumitru et al. (2016) suggested that a pro-environmental policy influences the electricity saving habit of employees [14]. Goal setting, combined with feedback to an employee, positively affects energy conservation behavior [30]. Handgraaf et al. (2013) found that the use of rewards can motivate the energy-saving behavior of employees [19]. Zhang et al. (2013) determined that an organizational electricity saving climate positively affects the personal norms and electricity saving behavior of employees [4]. Werner et al. (2012) demonstrated that a simple and short presentation is an effective means to encourage instructors to turn off the lights in empty classrooms [21]. 

Leadership is inevitable in various organizations. Most workers spend over 30% of their time performing interactive activities during a workday, and leaders are the major interactants and initiators in such processes [31]. They are expected to influence the electricity saving behavior of their subordinates through energy saving management. However, the aforementioned studies afforded minimal attention on the energy management of leadership. To fill in this gap, the current study adopted CLT from the organizational field to explore how charismatic leadership (CL) in energy management affects the energy saving behavior of employees. 

### 2.2. CLT 

Charismatic leaders are effective transmitters of values; they guide the behavior of their followers through shaping their intrinsic motivation, such as values, beliefs, responsibility and obligation [23,32]. CLT is widely adopted in the field of organizational behavior to explore how CL increases the intrinsic motivation of employees and then promotes their extra-role behaviors, such as organizational citizenship behavior (OCB) [23,33]. Extra-role behavior refers to a positive behavior of employees that is not included in their job specifications and is not directly rewarded, but remains beneficial for their organization [11]. Specifically, charismatic leaders transmit organizational vision to their employees. If the employees identify with the vision, they will have a sense of responsibility to undertake extra-behaviors for the benefit of their organization. Electricity saving behavior can be regarded as a kind of extra-role behavior because it is not a requirement for employees and will not be rewarded directly, but will benefit organizations by reducing operational costs [11]. Therefore it is reasonable to adopt CLT in exploring the electricity saving behavior of employees. Charismatic leadership is expected to evoke employees’ sense of responsibility in electricity saving (i.e., a kind of intrinsic motivation to save electricity), which in turn improves the electricity saving behavior of employees. 

CLT [34] indicates that leader charisma is formed based on four core components [23]: (1) envisioning (a charismatic leader shares a desirable goal (i.e., the vision) of organizational development); (2) behavioral cues (a charismatic leader identifies which tasks should be undertaken and how to perform them to realize the goal); (3) inspiration (a charismatic leader uses a powerful and interactive style to evoke the followers’ sense of responsibility in performing the tasks and realizing the goal); and (4) role modeling (as a role model, a charismatic leader sets an example for his/her followers and allows them to emulate his/her attitude and behavior on how to perform tasks and realize the goal). 

In accordance with CLT, the four factors are expected to shape the intrinsic motivation of employees [23,33]. In particular, a charismatic leader clearly articulates the goals of an organization (i.e., envisioning), which should also match the potential needs of followers, and thus the followers will shoulder the joint responsibility to realize the vision [23]. However, a vision can be vague. Behavioral cues, such as “what should be done” and “how to do it,” are vision implementation techniques and should be required to improve the practicability of a vision. Consequently, the practicability of organizational vision should increase the identification of employees with the organizational value [23,33]. Then, inspirational communication demonstrates that the leader is energized by the vision and calls for joint efforts of his/her followers, which should increase employee sense of responsibility to input effort for realizing the vision [23]. Finally, if the followers regard the leader as a role model, they will learn and emulate how the leader takes responsibility for realizing the vision [23,33]. Consistent with these arguments, researchers found that charismatic leadership produces high identification among followers, strong emotional attachment to the leader and attitudinal congruence between leaders and employees [32]. 

The underlying assumption of CLT is that employee behaviors are influenced by the personal charisma of their leaders through shaping their intrinsic motivation [23,34]. It neglects organizational policy, reflecting formal authority, which affects employee behavior through shaping their extrinsic motivation. An employee, as a member of an organization, cannot go beyond the effect of formal policy [35]. To better understand electricity saving behavior in workplaces, it is necessary to consider both the informal influence of leader charisma and the formal influence of organizational reward policy. In this study, we test how organizational reward policy on electricity saving affects employee electricity saving behaviors. 

## 3. Research Model and Hypotheses

### 3.1. Research Model 

**Figure 1:**
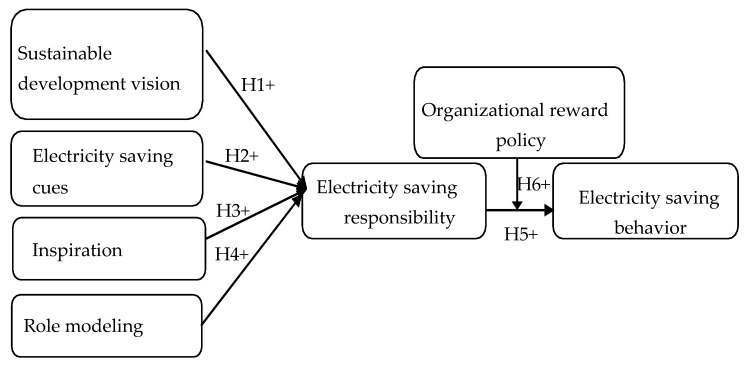
Research model.

Figure 1 presents the research model, which aims to elucidate electricity saving behavior in the workplace. The four characteristics of charismatic leadership, namely envisioning, behavioral control, inspiration, and role modeling, are expected to influence the energy saving responsibility of employees. This intrinsic motivation, along with organizational reward policy (i.e., a stimulus of extrinsic motivation), is regarded as a predictor of the electricity saving behavior of employees. Demographic variables (i.e., age, gender, income, education, industry, and tenure) are set as the control variables. 

### 3.2. The Predictors of Electricity Saving Responsibility 

An individual’s electricity saving responsibility refers to an individual’s sense of responsibility regarding whether people should save electricity and how much of an effort he/she should exert in order to do so [36]. It is an intrinsic motivation and is expected to be inspired by leader charisma [23]. First, charismatic leaders can articulate a sustainable development vision of electricity saving that matches the potential needs of their subordinates [23]. The vision illustrates how the electricity saving behavior of an employee helps reduce carbon emission and preserve a better ecological environment for future generations. This vision fits into the personal needs of an employee given that his/her descendants will live in such an ecological environment. Moreover, the vision should demonstrate how the electricity saving behavior of an employee helps his/her organization reduce consumption cost and achieve a “green” public image. The latter indirectly enhances the self-image of the employee as someone who is working for an organization with “good public image” [16,35]. The vision of sustainable development can be effective in evoking the sense of responsibility of employees because it reflects a variety of desirable outcomes from electricity saving activities that indirectly satisfy the personal needs of employees. The vision is inconsistent with the status quo but can be possibly realized through the electricity saving behavior of employees [23], and thus can improve their willingness to engage in electricity saving activities. The preceding arguments are summarized in the following hypothesis:

**Hypothesis 1** **(H1).** *Sustainable development vision is positively related to the electricity saving responsibility of employees.*

Visions are vague and charismatic leaders do not simply articulate a vision, but also clarify electricity saving cues which refer to the implementation of electricity saving techniques [23]. In the current study, electricity saving cues include “What electrical equipment can be used to save electricity in the workplace?” and “How do we save electricity when using such equipment?” Electricity saving cues tell employees “what to do” and “how to do it” when engaging in electricity saving activities. They increase the perceived behavioral control of employees (i.e., having opportunities and capabilities to save electricity) [28]. If employees perceive that they have more opportunities and capabilities to save electricity, they will be more willing to take responsibility. In line with this argument, Siero et al. (1989) reported qualitative findings that if office workers lack knowledge about how to perform energy conservation behaviors, they will think they are not able to take responsibility for energy conservation [37]. Thus, we propose the following hypothesis:

**Hypothesis 2** **(H2).** *Electricity saving cues are positively related to the electricity saving responsibility of employees.*

The third dimension of leader charisma is manifested in inspirational communication. Inspiration means that charismatic leaders speak with confidence and demonstrate a powerful interaction style, such as having direct eye contact, animated facial expressions, and a captivating voice tone, to disseminate the effectiveness of electricity conservation in realizing the sustainable development vision [23]. Inspiration is effective as it reflects leaders’ powerful nonverbal tactics. Through inspirational communication, leaders emphasize employee joint responsibility in electricity saving and demonstrates their confidence in the effectiveness of electricity saving to realize sustainable development. These characteristics will increase employee perceived value and the significance of electricity saving in the workplace. As a result, their sense of responsibility in electricity saving is increased. Thus we hypothesize:

**Hypothesis 3** **(H3).** *Inspiration is positively related to the electricity saving responsibility of employees.*

Role modeling refers to charismatic leaders acting as role models to propagate their electricity saving awareness and to set a good example of electricity conservation for their employees, thereby encouraging the inner motivation of employees to emulate and follow them [38]. Employees who identify with their charismatic leaders will attempt to learn the attitude and behavior of their leaders because they believe that their leaders are more knowledgeable than they are. The desire to learn from their charismatic leaders will motive employees to take responsibility for electricity saving.

**Hypothesis 4** **(H4).** *Role modeling is positively related to the electricity saving responsibility of employees.*

The sense of responsibility of employees is the precondition for their electricity saving behaviors [36]. Electricity saving responsibility can be deemed as an intrinsic motivation, which involves the employee participating in electricity saving activities for some sort of internal obligation. Extra-role behaviors are often inspired by individual intrinsic motivations [11], and therefore electricity saving responsibility is expected to inspire electricity saving behavior in employees [4]. On the contrary, if employees feel that they assume no responsibility for electricity saving, they will not perform their obligation by undertaking electricity saving behaviors. In line with the above arguments, Zhang et al. (2013) found that electricity saving responsibility positively affects employee sense of obligation, which in turn improves the electricity saving behavior of employees [4]. Moreover, Gadenne et al. (2011) found that general environmental beliefs, a concept similar to electricity saving responsibility, have a positive relationship with environmental behavior attitudes and environmental behaviors [35]. Thus, the above arguments are captured in the following hypothesis:

**Hypothesis 5** **(H5).** *Electricity saving responsibility is positively related to the electricity saving behavior of employees.*

Prior literature shows that individual behaviors are stimulated by two classes of motivation: extrinsic and intrinsic. Intrinsic motivation refers to internal needs/satisfaction involving an individual’s undertaking of a behavior. Extrinsic motivation pertains to external rewards/goals driving an individual to perform a behavior [27]. In reality, organizations may take interventions to drive electricity saving behavior of employees by shaping their intrinsic and extrinsic motivations. In extant literature, several researchers investigated the main effects of the two types of motivations on electricity saving behavior of employees [4,11,16,28,39]. They found that intrinsic motivation (e.g., enjoyment) significantly improved the electricity saving behavior of employees [4,16]. However, the effect of extrinsic motivation (e.g., organizational reward) on electricity saving behavior was ambiguous. For instance, Zhang et al.’s (2014) empirical findings show that organizational reward has no significant effect on the electricity saving behavior of employees [16]. However, Locke (1968) posited that organizational reward plays an important role in determining the energy saving attitude of employees [39]. The inconsistent findings suggest that there is a need to investigate whether extrinsic motivation interacts with other factors to affect employee energy saving behaviors.

Given that many managers tend to drive the two types of motivations simultaneously, it is interesting to explore how employee intrinsic motivation interacts with extrinsic motivation to affect electricity saving behaviors. CLT highlights that leaders inspire employee electricity saving behavior by shaping their intrinsic motivations (i.e., a sense of responsibility), but does not consider the effect of their extrinsic motivations. To fill this gap, this study investigates how organizational reward policy, as a typical stimulus of extrinsic motivation, interacts with electricity saving responsibility to affect electricity saving behaviors.

Organizational reward policy refers to the policy made by an organization to reward the electricity saving behavior of employees with bonuses, promotion, training opportunities, etc. [16,35]. When the electricity saving behavior of employees is extrinsically motivated by rewards, their attention will focus more on the rewards. Their colleagues will perceive that they save electricity because of the rewards [40]. These negative perceptions will be more likely to make the employees attribute their electricity saving behaviors to the motivation of obtaining rewards, instead of believing that they do this because of their sense of responsibility. In other words, organizational rewards crowd out the effect of a sense of responsibility on electricity saving behavior [40]. By contrast, when organizations have no policies to reward electricity saving behavior, employees will attributes their electricity saving behaviors to their intrinsic motivations. Then, an inner sense of responsibility becomes a strong factor influencing electricity saving behavior. Thus, we propose the following hypothesis:

**Hypothesis 6** **(H6).** *The positive relationship between employees’ responsibility of electricity saving and electricity saving behavior is weaker when organizational reward is high than when it is low.*

## 4. Research Methodology

### 4.1. Measures

We adapted from or developed the measurement of the studied variables according to prior literature (described below). The questionnaire was translated into Chinese by means of a back-translation procedure [41], so that the survey could be conducted in Chinese. We used short scales to reduce fatigue among respondents, and the measures of the studied variables included 23 questions. All items were measured by a five-point Likert scale ranging from 1 = strongly disagree to 5 = strongly agree. Specifically, three items for sustainable development vision were adapted from Griffin et al. (2010) [42]. The scales of electricity saving cues and inspiration were adapted from Kirkpatrick and Locke (1996) [23]. Role modeling was measure by a self-developed scale based on the theoretical research of role modeling [38]. The measure of electricity saving responsibility was adapted from De Groot and Steg (2009) [36]. The scale of organizational reward policy was adapted from Bock et al. (2005) [43]. Electricity saving behavior was measured by an seven-item scale adopted from Zhang et al. (2013) [4]. Table 1 lists the final items used in the questionnaire.

Two professors and one Ph.D student in the fields of energy conversation and organizational behavior were invited to discuss the items. A minor revision has been made on the questionnaire according to their feedback.

### 4.2. Data Collection Procedure

An online questionnaire was released on two professional survey websites which had two nationwide sample databases consisting of more than 5.2 million online members who might participate in the online surveys released on the two websites. In exchange for their participation, monetary reward was provided for each participant. The two websites recorded 1886 visits to our questionnaire. We used a filtering question at the beginning of the survey to identify respondents who matched our targeted profile of full-time office workers working in offices of various organizations. Of the 1886 visitors, 627 satisfied our sampling criteria, representing a response rate of 33%. The respondents were from 14 provinces or municipalities of China, including Beijing, Shanghai, Anhui, Zhejiang, Tianjin, Henan, Jiangsu, Guangdong, Shandong, Jiangxi, Fujian, Hubei, Liaoning, and Hebei. Table 2 presents their demographic information.

## 5. Data Analysis and Findings

Validity and reliability of the variables were assessed by LISREL (version 8.70), using Confirmatory Factor Analysis (CFA) [44], and SPSS V20. Hypotheses were tested by SPSS V.20, using multiple linear regression analysis, moderated multiple regression analysis and Hayes’ (2013) bootstrapping approach [45].

### 5.1. Measurement Model

The Cronbach’s alpha of all studied variables ranged from 0.73 to 0.82, demonstrating that the reliability of each scale was within the acceptable limit (0.70) (see Table 3). CFA was conducted to assess the validity of all measures. All loadings were significant (*p* < 0.01) (see Table 3). Loadings from 0.50 to 0.55 were considered fair; 0.63 to 0.70 very good; and above 0.71 excellent [46,47]. The results indicate that the reliability and convergent validity of the measures are satisfactory. The variance inflation factor (VIF) values for all studied variables are all below 5 threshold [48], representing that the dataset has no multicollinearity problem.

Secondly, CFA was conducted to assess the overall goodness of fit of the measurement model. We used five model-fit indices: the ratio of x^2^ to degrees of freedom (df), Non-Normed Fit Index (NNFI), comparative fit index (CFI), Goodness of Fit Index (GFI), and root mean square error of approximation (RMSEA). The measurement model, containing seven factors (i.e., vision, electricity saving cues, inspiration, role modeling, electricity saving responsibility, organizational reward policy, and electricity saving behavior) yielded a good fit (x^2^ = 767.11; df = 209; *p* < 0.005; GFI = 0.90, NNFI = 0.96, CFI = 0.96, RMSEA = 0.065). The results show that the measurement model fits the data very well.

In addition, the chi-square difference test [49] was adopted to compare the seven-factor model with the one-factor model. The results show that the fit of the seven-factor model is significantly better than one-factor model. The results not only demonstrate that all measures have good discriminant validity and exhibit a good fit with the data, but also minimize the possibility of common method bias [50].

### 5.2. Results of Hypotheses Testing

To test Hypothesis 1–4, a multiple linear regression analysis was conducted. Table 4 summarizes the research findings. Altogether, the model explained 21% of the variance in electricity saving responsibility. All of the four hypotheses were supported. Hypothesis 1 posits that sustainable development vision improves electricity saving responsibility. Results show that the coefficient of this relationship is 0.21 (*p* < 0.01). Hypothesis 2 states that electricity saving cues increase electricity saving responsibility. Results show the path coefficient is 0.15 (*p* < 0.01). Hypothesis 3 proposes that inspiration improves electricity saving responsibility, and research findings demonstrate the coefficient of this relationship is 0.13 (*p* < 0.01). Finally, hypothesis 4 posits that role modeling increases electricity saving responsibility, and results show the path coefficient is 0.12 (*p* < 0.01). Thus, Hypotheses 1–4 were supported. A moderated multiple regression was used to test Hypothesis 5–6. Results are depicted in Table 4. As expected, electricity saving responsibility and organizational reward policy are positively related to electricity saving behavior (β = 0.50, *p* < 0.01; β = 0.40, *p* < 0.01). Hypothesis 5 was supported. The relationship of electricity saving responsibility with electricity saving behavior is negatively moderated by organizational reward policy (β = −0.11, *p* < 0.01). The positive relationship of electricity saving responsibility with electricity saving behavior is weaker when organizational reward is high than when it is low. Thus, hypothesis 6 was supported.

Moreover, we conducted post-hoc analysis by using Hayes’ (2013) bootstrapping approach (n boots = 1000; 95% bias corrected confidence interval) to test the mediation (i.e., indirect effect). Bootstrapping was found to be the most powerful method to detect mediation or indirect effects [45]. It has been used by many studies in the fields of organizational behavior, psychology, and information systems [51]. A confidence interval must not contain a zero to assume a significant mediation or conditional indirect effect [45].

Results are presented in Table 5. The bootstrapping analysis found that sustainable development vision exerted a positive and indirect effect on electricity saving behavior though electricity saving responsibility (β = 0.10, *p* < 0.05) [(BC 95% CI); 0.06, 0.15]. The indirect effect of electricity saving cues on electricity saving behavior though electricity saving responsibility is also positive and significant (β = 0.09, *p* < 0.05) [(BC 95% CI); 0.06, 0.13]. Likewise, inspiration has a positive and indirect effect on electricity saving behavior though electricity saving responsibility (β = 0.10, *p* < 0.05) [(BC 95% CI); 0.06, 0.14]; and role modeling has a positive and indirect effect on electricity saving behavior though electricity saving responsibility (β = 0.06, *p* < 0.05) [(BC 95% CI); 0.03, 0.09].

## 6. Discussion and Implications

This study adopted CLT to examine how the four characteristics of charismatic leadership shape the electricity saving responsibility of employees, which in turn increases their electricity saving behaviors in the workplace. This research model also examined the moderating role of organizational reward policy in electricity conservation. Previous research on CLT has focused mainly on the field of organizational behavior. The current study is the first to apply and confirm CLT in the context of energy conservation in China. All the hypothesized relationships are supported.

### 6.1. Theoretical Implication

This research contributes to the energy conservation literature in several aspects. First, existing studies on energy conservation focus on household electricity conservation and the electricity conservation behavior of employees has received limited attention [4]. However, organizations consume more electricity than households. Compared with household electricity saving behavior, promoting the electricity saving behavior of employees in organizations is more difficult [8]. This study contributes to the literature by establishing a theoretical model for exploring the electricity saving behavior of employees in the workplace. Our research findings provide important insights and advance the understanding of the electricity saving behavior of employees.

Secondly, our research findings confirm the rationale of CLT [23,32] and demonstrate that this theory can be adopted to explain the energy conservation phenomenon in the context of the workplace. Extant energy conservation literature has explored the effect of a series of organizational factors on the electricity saving behavior of employees, but has disregarded the effect of leadership, which is an indispensable organizational factor in the context of the workplace [8]. On the basis of CLT, the present study identifies the four characteristics of charismatic leadership that are positively relate to electricity saving motivation and behavior, and thus introduces four new concepts, namely, vision, electricity saving cues, inspiration, and role modeling, as new predictors in the energy conservation literature. Moreover, this study explores the underlying processes through which charismatic leadership affects electricity saving behavior. In this manner, this study establishes a bridge between the charismatic leadership literature and the energy conservation literature.

Thirdly, although prior studies investigated the main effects of both employee intrinsic motivation and extrinsic motivation on their electricity saving behaviors [11,16], research on the interactive effect of the two types of motivations is lacking. Perhaps, in real-world organizations, managers tend to improve both types of motivation, as both of them are assumed to be beneficial. To address this gap, the novel finding of our studies shows that organizational reward policy undermines the positive effect of electricity saving responsibility on electricity saving behavior. This result not only introduced a new contextual factor to energy conversation literature but provided a deeper understanding of the complex interaction between intrinsic and extrinsic motivations in electricity saving behavior.

### 6.2. Practical Implication

Our research also has clear practical implications for electricity saving management within organizations. First, employees’ sense of responsibility for electricity saving should be the focus of training, as it is an important intrinsic motivation that effectively influences the energy saving behavior of employees. Second, leader charisma is useful in cultivating the energy saving responsibility of employees. Specifically, sustainable development vision can be set up. The vision should identify sustainable development goals with personal goals of employees, and then managers can propagate the vision to drive employees’ sense of responsibility in energy saving. Subsequently, managers should provide implementation techniques for energy saving to guide employee behaviors. These implementation techniques can be delivered by professional training, pro-environmental seminars, or leaflets. Managers should also improve their inspiration communication skills to encourage employees in actively taking responsibility for energy saving. Moreover, it is very important for managers to set a good example as “green leaders” and show how they perform their responsibility for energy saving. The energy saving behavior of leaders should gain publicity through various information channels, such as organizational meetings, internal websites and internal newspapers. What deserves attention is that, if charismatic leadership is adopted in energy saving management within an organization, organizational reward policy for energy saving will become redundant. Although both may improve the energy saving behavior of employees, managers need not use the two stimuli simultaneously, as one should offset the positive effect of the other on the energy saving behavior of employees.

### 6.3. Limitations

We acknowledge that this study has limitations. First, it focuses on electricity conservation in organizations. Various types of organizations have different ownerships, and exploring whether the electricity saving behavior of employees in state-owned enterprises differs from those of employees in private enterprises will be interesting. Future studies can use the ownership of organizations as a moderating variable in our theoretical model to explore whether it moderates the effect of CL on the electricity saving behavior of employees. Second, the data are based on self-reports, and the common method variance problem may pose a validity threat. However, confirmatory factor analysis shows that the items exhibit good discriminant validity, thereby suggesting that the effect of the common method variance is insignificant [50]. Nonetheless, future research should include data from different sources to address this issue. Third, future research should further test our model using longitudinal data because the causal relationships implied in our reasoning cannot be ascertained in a cross-sectional design. Fourth, only a Chinese sample was used, which suggests that the framework proposed in the present study may be limited to applications in Chinese culture. Future researchers should conduct a cross-cultural study to re-examine the universality of our research model. Fifth, this study only takes office workers as a sample, and thus we should take caution when generalizing the research findings to samples of other types of workers (e.g., production workers). Future researchers could explore the electricity saving behavior of other types of workers.

## 7. Conclusions

This study developed and empirically tested a research model that explained the predictors of the electricity saving behavior of employees based on CLT and individual motivation literature. Data were collected by conducting surveys among employees in 14 provinces or municipalities of China. SPSS and LISREL were used to analyze the data and test the hypotheses. The research findings show that organizational reward policy for electricity conservation exerts a negative moderating effect on the relationship between electricity saving responsibility and electricity saving behavior. Sustainable development vision, electricity saving cues, inspiration, and modeling positively influence electricity saving responsibility. This research provides insights into improving electricity saving behavior in organizations. This study has several limitations and suggests future directions of study. For instance, we do not include ownership of organizations, which may be an important predictor of employees’ electricity saving behavior, and therefore we call for future studies to test the moderating effect of this variable in our study model. Moreover, our data is self-reported and cross-sectional. Future research can use longitudinal data from different sources to re-examine our research framework. Further, only a Chinese sample was used in this study, and thus we call for a cross-cultural study to re-examine the universality of our research findings. The last point is that our sample consists of office workers and future researchers should focus on other types of workers to improve results generalizability.

## Figures and Tables

**Table 1:** Measurement items.

Construct	Survey Questions
Sustainable development vision	1. My leader describes an exciting and attractive image of our sustainable development which can be realized through our energy saving behaviors.
	2. My leader has a clear understanding of the direction of our sustainable development which can be realized through our energy saving behaviors.
	3. My leader expresses a clear goal of our sustainable development which can be realized through our energy saving behaviors.
Electricity saving cues	1. My leader lets me know which electrical equipment can be used as a way of saving electricity.
	2. My leader gives me suggestion on effective methods to save electricity when using this electrical equipment.
Inspiration	1. My leader heightened my motivation to save electricity.
	2. My leader aroused in me the effort to save electricity.
	3. My leader increased my optimism about the development of our organization if I participate in electricity saving activities.
Role modeling	1. My leader sets a good example in saving electricity for our organization.
	2. My leader is concerned how his/her electricity saving attitude and behavior affects us.
Organizational reward policy	1. My organization provides rewards in return for my electricity saving behavior.
	2. My organization has a policy to reward our electricity saving behaviors.
Electricity saving responsibility	1. I feel jointly responsible for saving electricity.
	2. I feel joint responsibility for saving electricity to avoid global warming.
	3. I feel joint responsibility for saving electricity to reduce local ecological damage.
	4. I feel joint responsibility to avoid exhaustion of electricity.
Electricity saving behavior	1. I often turn off the lights in my workplace when going out even for a short time.
	2. I reduce the use of the air conditioner by opening the windows in my workplace.
	3. I switch off the computer in my workplace when it is not used.
	4. I shorten the duration that the refrigerator door is kept open in my workplace.
	5. I turn off the lights in my workplace when the sunshine is bright enough.
	6. I properly close the room when I use the air-conditioner in my workplace
	7. I switch off all lights when leaving my workplace as the last person.

**Table 2:** Demographics summary.

Tenure	Percentage (%)	Gender	Percentage (%)
<0.5	1.3	Male	45.3
0.5–1	2.7	Female	54.7
1–3	24.4	Education	
4–6	31.3	Secondary School	1.6
7–9	19.9	College	15.2
≥10	20.4	Bachelor	75.0
Income		Master or above	8.3
2000–5000 RMB	15.6	Industry	
5001–10,000 RMB	53.4	Manufacturing	30.0
10,001–15,000 RMB	22.3	Finance	8.5
15,001–20,000 RMB	5.7	Real estate	4.0
>20,000 RMB	2.9	Architecture	5.9
		Medical care	11.8
Age		Education	5.6
20–29	31.3	Information Technology	13.2
30–39	54.1	Wholesale & Retail	6.7
40–49	11.2	Transportation &Storage	5.3
≥50	3.5	Mining	1.0
		Others	8.1

**Table 3:** Results of the validity and reliability analysis.

Construct	Cronbach’s Alpha	VIF	Items	Loading
Sustainable development vision	0.82	2.121	Vision1	0.80
			Vision2	0.77
			Vision3	0.76
Electricity saving cues	0.73	1.856	Cues1	0.76
			Cues2	0.76
Electricity saving responsibility (ESR)	0.80	1.600	ESR1	0.69
			ESR2	0.74
			ESR3	0.75
			ESR4	0.66
Inspiration (INS)	0.81	1.945	INS1	0.78
			INS2	0.76
			INS3	0.75
Role modeling (RM)	0.80	1.088	RM1	0.68
			RM2	0.98
Electricity saving behavior (ESB)	0.73	2.001	ESB1	0.55
			ESB2	0.51
			ESB3	0.50
			ESB4	0.55
			ESB5	0.58
			ESB6	0.54
			ESB7	0.56
Organizational reward policy (ORP)	0.76	1.891	ORP1	0.83
			ORP2	0.75

**Table 4:** Results of hypothesis testing.

	Electricity Saving Responsibility
	Standardized Coefficient (β)
Sustainable development vision	0.21 **
Electricity saving cues	0.15 **
Inspiration	0.13 **
Role modeling	0.12 **
*R* ^2^	0.21 **
	Electricity saving Behavior
	β
Electricity saving responsibility (ESR)	0.50 **
Organizational reward policy (ORP)	0.40 **
ESR	0.46 **
ORP	0.41 **
ESR * ORP	−0.11 **
*R* ^2^	0.47 **

Note. * *p* < 0.05; ** *p* < 0.01.

**Table 5:** Results of testing indirect effects (Mediation).

Indirect Effects	BC 95% CI			
	β	SE	Lower	Upper
Vision →Responsibility →ESB	0.10	0.02	0.06	0.15
Cues →Responsibility →ESB	0.09	0.02	0.06	0.13
Inspiration →Responsibility →ESB	0.10	0.02	0.06	0.14
Role modeling →Responsibility →ESB	0.06	0.01	0.03	0.09
